# Supplementary figures and images for: Comparison of radiomics-based machine-learning classifiers for the pretreatment prediction of pathologic complete response to neoadjuvant therapy in breast cancer
Source: PeerJ. 2024 Jul 15;12:e17683. doi: 10.7717/peerj.17683 (PMC11257043; doi:10.7717/peerj.17683)

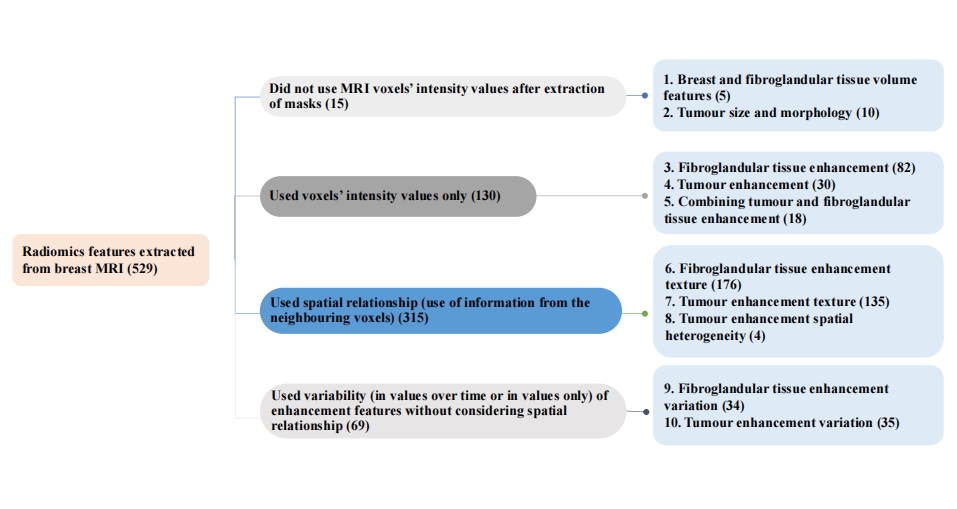

Supplement: Supplemental Information 1 [file peerj-12-17683-s001.jpg]
